# Genetic Effects Conferring Heat Tolerance in a Cross of Tolerant × Susceptible Maize (*Zea mays* L.) Genotypes

**DOI:** 10.3389/fpls.2016.00729

**Published:** 2016-06-02

**Authors:** Muhammad Naveed, Muhammad Ahsan, Hafiz M. Akram, Muhammad Aslam, Nisar Ahmed

**Affiliations:** ^1^Department of Plant Breeding and Genetics, University of AgricultureFaisalabad, Pakistan; ^2^Pulses Research Institute, Ayub Agricultural Research InstituteFaisalabad, Pakistan; ^3^Plant Physiology Section, Agronomic Research Institute, Ayub Agricultural Research InstituteFaisalabad, Pakistan; ^4^Centre of Agricultural Biochemistry and Biotechnology, University of AgricultureFaisalabad, Pakistan

**Keywords:** heat tolerance, genetic basis, physiological traits, yield components, maize

## Abstract

Incessant rise in ambient temperature is threatening sustainability of maize productions, worldwide. Breeding heat resilient synthetics/hybrids is the most economical tool while lack of knowledge of gene action controlling heat and yield relevant traits in maize is hampering progress in this regard. The current study, therefore, was conducted using analyses of generation mean and variance, and narrow sense heritability (${\text{h}}_{\text{n}}^{2}$) and genetic advance as percent of mean (GAM%). Initially, one hundred inbred lines were evaluated for cell membrane thermo-stability and grain yield per plant on mean day/night temperatures of 36.6°C/22.1°C in non-stressed (NS) and 42.7°C/25.7°C in heat-stressed (HS) conditions. From these, one tolerant (ZL-11271) and one susceptible (R-2304-2) genotypes were crossed to develop six basic generations, being evaluated on mean day/night temperatures of 36.1°C/22.8°C (NS) and 42.3°C/25.9°C (HS) in factorial randomized complete block design with three replications. Non-allelic additive-dominance genetic effects were recorded for most traits in both conditions except transpiration rate, being controlled by additive epistatic effects in NS regime. Dissection of genetic variance into additive (D), dominance (H), environment (E) and interaction (F) components revealed significance of only DE variances in HS condition than DE, DFE and DHE variances in NS regime which hinted at the potential role of environments in breeding maize for high temperature tolerance. Additive variance was high for majority of traits in both environments except ear length in NS condition where dominance was at large. Higher magnitudes of $\sigma_{\text{D},}^{2}$ ${\text{h}}_{\text{n}}^{2}$ and GAM% for cell membrane thermo-stability, transpiration rate, leaf firing, ear length, kernels per ear and grain yield per plant in both regimes implied that simple selections might be sufficient for further improvement of these traits. Low-to-moderate GAM% for leaf temperature and 100-grain weight in both conditions revealed greater influence of genotype-environment interactions, indicating ineffective direct selection and advocating for further progeny testing. In conclusion, pyramiding of heritable genes imparting heat tolerance in maize is achievable through any conventional breeding strategy and generating plant material with lowest cellular injury and leaf firing, and higher transpiration rate, ear length, kernels per ear and grain yield per plant.

## Introduction

Climate change and agriculture are interlinked and affect each other (Hoffmann, 2013). Global warming, being the major cause of climate change, is increasing the concentrations of atmospheric greenhouse gases (GHGs) which slowly but gradually are heating up earth's temperature (Treut et al., 2007; IPCC, 2014). Increase in temperature beyond optimum may cause shifting of agricultural lands and shortening of cropping periods (Porter, 2005). Heat stress is a worldwide agricultural issue that can induce anatomical, biochemical and morpho-physiological alterations in crop plants resulting in heavy production losses (Wahid et al., 2007). It affects plant development right from germination till final harvest. Short term effects of high temperature stress may either be cellular injury or cell death due to increased ion leakage caused by denaturation of membrane proteins or increased fluidity of membrane lipids. However, long term impact may be decrease in size of cells, tissues and organs, thus, hampering plant growth (Schöffl et al., 1998; Savchenko et al., 2002).

Being a member of C_4_ plant kingdom, high temperature has both favorable and unfavorable effects on maize crop. Optimum day (25–32°C) and night (16.7–23.3°C) temperatures for maize plant lead to enhanced photosynthetic rate than respiration resulting in rapid plant growth. However, plant growth affected severely when optimum temperature decreases to 5°C or increases beyond 32°C (Steven et al., 2002). Detrimental effects of heat stress include malfunctioning of reproductive organs (desiccation of silk and pollen grains, reduced pollen germination, increased flower abortion, fertilization failure and shrunken seeds), photosynthetic acclimation and disrupting other physiological processes directly and changing the pattern of plant development indirectly (Sinsawat et al., 2004; Kim et al., 2007; Ristic et al., 2009). Once pollination is accomplished, then developing kernels depend entirely on the source of photosynthates. Rise in temperature beyond 30°C impacts the activity of *Rubisco* in maize, which in turn reduces photosynthesis and ultimately decreases grain filling period and grain size (Steven et al., 2002). A temperature of 35°C during pollination and grain filling stages may reduce grain yield on a daily basis by 101 kg ha^−1^ (Smith, 1996). Likewise, an increase in mean daily temperatures from 22 to 28°C during the grain filling period may cause 10–42% yield losses (Lobell and Burke, 2010; Rowhani et al., 2011; Cairns et al., 2013).

Heat tolerance can be accomplished through genetic management approach. Development of stress tolerant varieties would be a cheap input technology that would play a vital role in lessening the harmful impacts of abiotic stresses on agricultural production (Saxena and O'Toole, 2002; Tester and Langridge, 2010). As heat tolerance is a quantitatively inherited trait and is more prone to genotype-environment interactions, therefore building resistance against it, is a complex task. Identification of superior genetic resources and introgression of intended genes in promising genotypes are the primary steps involved in the development of any genetic management technology (Chen et al., 2012). Prior to gene transfer, an understanding of genetic effects involved in inheritance of various morphological and physiological parameters controlling heat tolerance in the genetic material, being researched, is necessary to divert efforts in that direction and to formulate effective selection criteria to accomplish this goal. Literature pertaining to gene action controlling important plant traits of maize under heat stress is scanty. In maize, both additive and dominance gene actions control inheritance pattern of leaf temperature (Hussain et al., 2009), cell membrane thermo-stability (Saleem et al., 2015), leaf firing (Kaur et al., 2010), ear length (Ahmed et al., 2000), kernels per ear (Muraya et al., 2006), 100-grain weight (Wu, 1987) and grain yield per plant (Iqbal et al., 2007). However for transpiration, non-additive genetic effects are more crucial in comparison to additive gene action (Akbar et al., 2009). A few researchers also reported contrasting genetic effects for these traits (Tabassum and Saleem, 2005; Kumar and Sharma, 2007; Kanagarasu et al., 2010). Various biometrical techniques could be used for appraising gene action. Among these, generation mean analysis (GMA) is the most widely used which provides information not only on presence or absence of epistasis, but also determines both additive and dominance variances and effects (Singh and Narayanan, 1993). The present research work, therefore, was undertaken to assess the genotypic variation for heat tolerance in maize, identifying two most contrasting genotypes for developing plant material in order to assess genetic effects involved in inheritance of various metric traits in basic six generations (P_1_, P_2_, F_1_, F_2_, BC_1_, and BC_2_) under heat-stressed and non-stressed environments.

## Materials and methods

In order to explore the potential and genetics of heat tolerance in maize, research works were conducted during spring (February-April) and autumn (July-October) seasons of 2012–2015 in Pakistan at the experimental sites of Department of Plant Breeding and Genetics, University of Agriculture, Faisalabad (31.43° N, 73.06° E) and Plant Physiology Section, AARI, Faisalabad (31.25° N, 73.09° E).

### Experiment 1: Screening for identification of parents

During February 2012, one hundred maize inbred lines collected from various sources (**Table 2**) were sown in two sets, concurrently. Layout design used was alpha lattice (10 × 10) with 10 blocks, each comprising a total of 10 entries. Randomization in all the three replications was done using ALPHA software (Barreto et al., 1991). Row-to-row and plant-to-plant distances maintained were 75 and 25 cm, respectively using manual seed dibblers @ two seeds per hole which were thinned to one healthy seedling preceding 7 days after germination. The plot size measured for each entry was 2.81 m^2^, accommodating a total of fifteen plants in a single replication. All the recommended cultural practices comprising irrigations, fertilizers, insecticides were applied as and when required for both the treatments (Arain, 2013). Out of the two experimental sets, one was raised completely inside the tunnel (heat-stressed) while the other in open field (non-stressed) conditions. The set of inbred lines sown in the tunnel was exposed to high temperature stress by covering it with plastic sheet just prior to the onset of reproductive period upto the crop maturity (whole months of April and May). For recording the observations on different parameters, ten guarded plants were selected in a replication of each set. The humidity inside the plastic tunnel was controlled by exhaust fans to avoid any possible disease outbreak. The temperature recorded in non-stressed (NS) and heat-stressed (HS) environments is given in Table 1.

**Table 1:** **Temperature prior to the initiation of reproductive phase upto physiological maturity of maize crop (months of April and May)**.

**Temperature (°C)**		**Screening trials 2013**		**Evaluation trials 2015**	
		**Non-stressed**	**Heat-stressed**	**Non-stressed**	**Heatstressed**
Minimum	Range	14.0–30.8	24.2–31.4	15.6–32.2	18.3–34.3
	Mean	22.1	25.7	22.8	25.9
Maximum	Range	28.5–46.1	36.8–50.7	25.2–45.5	31.6–50.4
	Mean	36.6	42.7	36.1	42.3

### Appraisal of plant traits

Cell membrane thermo-stability (%) and grain yield per plant (g) were measured in screening phase, while, leaf temperature (°C), transpiration rate (μg cm^−2^ S^−1^), cell membrane thermo-stability (%), leaf firing (%), ear length (cm), kernels per ear, 100-grain weight (g), and grain yield per plant (g) were appraised in evaluation phase of experiment.

Cell membrane thermo-stability (CMT) was measured from non-stressed and heat-stressed experiments using the procedure of Sullivan (1972). With a punch machine, round leaf discs of 0.75 cm in diameter were made after removing completely expanded uppermost leaves. In two sets of 50 ml glass tubes, 10 leaf discs were taken and washed slowly with de-ionized distilled water by changing it three times to remove surface adhered electrolytes. Then glass tubes were filled up to 10 ml of distilled water in order to submerge the washed leaf discs. Of the two sets, one set of test tube was placed in a water bath at 45°C for 1 h. Both sets were then exposed to 22°C temperature in an air conditioned room for an overnight. Very next day, electrical conductivity of each test tube sample was recorded with the help of LF 538 EC meter after shaking it well. To kill the leaf tissues, both sets of test tube samples were autoclaved at 121°C temperature for 15 min at 15 Ibs pressure, which were allowed overnight to cool down at 22°C temperature. Subsequently, electrical conductivity was recorded for second time. Under stress, the extent of membrane integrity permits a measure of membrane stability to electrolyte leakage. Relative cell injury percentage (RCI%), an appraisal of cell membrane thermo-stability was worked out by using 1st and 2nd electrical conductivity readings. T and C indicate electrical conductivity (EC) of heat-stressed and non-stressed sets of test tube, and subscripts 1 and 2 refer to 1st and 2nd EC readings, respectively.

$$\text{RCI}\%=[1-\{1-(T_{1}/T_{2})\}/\{1-(C_{1}/C_{2})\}]\times \;100$$

Leaf temperature (LT) was recorded between 13.00 and 15.00 h by using infrared thermometer (Raytek, Model Raynger® 3i). Transpiration rate (TR) was recorded with the help of porometer (Li Cor Steady State, Model Li 1600) which was adjusted to existing environmental conditions with prevailed temperature and light quantum using null gain adjustment (NGA) procedure. Leaf firing (LF) was worked out by scoring plant leaves showing heat burnt symptoms for each entry while percent leaf firing (LF%) was estimated by dividing no. of plants with leaf firing symptoms with total no. of plants multiplied by 100 (Bai, 2003). Ears length from the selected plants was measured in cm with the help of measuring tape (Stanley Fat, Model 33-725). For determining kernels per ear, kernels harvested from each ear of each selected plant were counted separately and averaged thereafter. For 100-grain weight (HGW), three samples each comprising randomly selected 100 grains from produce of each selected plant were weighed in grams with the help of an electronic balance (Adam, Model NBL 12001e). Average was worked out for each entry. Grain yield per plant (GYPP) was recorded in grams by weighing total produce (grains) of selected plants of each entry using an electronic balance (Adam, Model NBL 12001e). Average was computed for each entry for further use. Observations on physiological parameters like leaf temperature, cell membrane thermo-stability and transpiration rate were recorded from fully expanded upper most three leaves before the onset of reproductive stage while morphological traits such as ear length, kernels per ear, 100-grain weight and grain yield per plant were measured on plant basis at physiological plant maturity. Leaf firing was recorded 30 days after heat stress. Data measured for each trait in a replication was averaged before utilization in statistical analyses.

Screening of genotypes was based on parameters like cell membrane thermo-stability (%) and grain yield per plant (g). Accessions with lowest CMT (%) and highest GYPP (g) were regarded as heat tolerant and *vice versa*. Cell membrane thermo-stability, an important and reliable measure of heat tolerance, has been largely used in assorting genotypes for this purpose (Ristic et al., 1998; Chen et al., 2014). It is negatively associated with yield as heat tolerant genotypes are more stable and yield greater in comparison to heat susceptible accessions (Azhar et al., 2009). Therefore, in order to keep the selection criteria simple and effective, these two characters were measured. Selection of the parents was done keeping in view the actual performances of genotypes for CMT and GYPP in non-stressed and heat stressed conditions. Assessment based on absolute performances under contrasting environments has been used previously by various researchers for the identification of tolerant and susceptible genotypes (Azhar et al., 2005; Akhter et al., 2007; Iqbal et al., 2011). The recorded data were subjected to statistical analysis of variance technique to find out significant differences among the inbred lines (Steel et al., 1997). One way and two way variance analyses and scatter plots were generated for both non-stressed and heat stressed environments using MINITAB version 16.1.1 (Minitab., 2010) software.

### Experiment 2 and 3: Development of breeding populations

Two genotypes, No. 89 (ZL-11271) and No. 5 (R-2304-2) were selected based on distinctiveness in responses to high temperature stress. Inbred line ZL-11271 was designated as heat tolerant parent (P_1_) with minimum relative cell injury percentage (RCI %) and maximum grain yield per plant (GYPP) while R-2304-2 as heat susceptible parent (P_2_) with highest RCI (%) and lowest GYPP under both non-stressed and heat-stressed conditions. These two most contrasting genotypes were used for further development of genetic material. Falconer (1952) suggested that a parent or a trait measured under two environments will be considered as two instead of one. During July 2013, both tolerant and susceptible parents were sown under normal field conditions and crosses were attempted to obtain filial generation one (F_1_)or hybrid (ZL-11271 × R-2304-2) seed. One hundred F_1_ plants along with parents (P_1_, P_2_) were raised in field during the months of February and July, 2014. Fifty F_1_ plants were advanced by selfing to filial generation two (F_2_) while the other fifty F_1_ plants (twenty-five each) were crossed by pollinating with ZL-11271 (P_1_) and R-2304-2 (P_2_) to develop backcrosses, BC_1_ (F_1_ × ZL-11271) and BC_2_ (F_1_ × R-2304-2) generations, respectively.

### Experiment 4: Evaluation of genetic material

During February 2015, two sets each comprising same six basic generations (P_1_, P_2_, F_1_, F_2_, BC_1_, and BC_2_) were planted simultaneously in a plastic tunnel (heat-stressed) and normal field (non-stressed) conditions in a factorial randomized complete block design with three replications. Experimental methodologies followed were same as in screening experiment. Thirty plants were planted each for parents, F_1_ and sixty for back crosses while three hundred for F_2_ generation in a replication. For the purpose of recording the observations in each replication, randomly guarded 20 plants from non-segregating (P_1_, P_2_, and F_1_) populations while 30 plants from BC_1_, BC_2_, and 60 plants from F_2_ generations were selected each from non-stressed and heat-stressed treatments. Temperature data recorded is given in Table 2.

**Table 2:** **List of maize inbred lines evaluated in non-stressed and heat-stressed environments**.

**IL**	**Name**	**Source**	**IL**	**Name**	**Source**	**IL**	**Name**	**Source**
1	F-128	MRS	34	Q-66	UAF	67	Y-54	MMRI
2	F-187	MRS	35	Q-67	UAF	68	Y-81	MMRI
3	F101-7-2-6	MRS	36	N-18	UAF	69	Y-91	MMRI
4	F-160	MRS	37	N-48-94	UAF	70	Y-101	MMRI
5	**R-2304-2**	MRS	38	PB-77	UAF	71	Y-52	MMRI
6	F-110	MRS	39	PB-7-1	UAF	72	Y-93	MMRI
7	F-189	MRS	40	52-B4	UAF	73	Y-42	MMRI
8	F-122	MRS	41	53-AP1	UAF	74	Y-36	MMRI
9	F-153	MRS	42	53-P4	UAF	75	Y-9	MMRI
10	F-164	MRS	43	82-P1	UAF	76	Y-15	MMRI
11	F-163	MRS	44	20-P2-1	UAF	77	Y-26	MMRI
12	F-107	MRS	45	L5-1	UAF	78	Y-21	MMRI
13	OH-8	UAF	46	L7-2	UAF	79	Y-18	MMRI
14	OH-28	UAF	47	70-NO2-2	UAF	80	Y-11	MMRI
15	OH-33-1	UAF	48	150-P1	UAF	81	Y-41	MMRI
16	OH-41	UAF	49	150-P2-1	UAF	82	Y-63	MMRI
17	OH-54-3A	UAF	50	HY-7	UAF	83	Y-113	MMRI
18	W-64-SP	UAF	51	IC-654	UAF	84	Y-83	MMRI
19	W-64-TMS	UAF	52	JY-1	UAF	85	Y-53	MMRI
20	WM-13-RA	UAF	53	UM-2	UAF	86	Y-89	MMRI
21	WF-9	UAF	54	Q-88	UAF	87	VL-106	CIMMYT
22	WFTMS	UAF	55	A-427-2	UAF	88	ZL-11376	CIMMYT
23	W-187-R	UAF	56	A-495	UAF	89	**ZL-11271**	CIMMYT
24	W-10	UAF	57	A-509	UAF	90	VL-1012835	CIMMYT
25	WA-3748	UAF	58	A-521-1	UAF	91	VL-1032	CIMMYT
26	W-82-3	UAF	59	M-14	UAF	92	VL-1033	CIMMYT
27	K-55-TMS	UAF	60	A50-2	UAF	93	VL-108496	CIMMYT
28	G.P.F-9	UAF	61	A-239	UAF	94	VL0512386	CIMMYT
29	USSR-40	UAF	62	A-545	UAF	95	VL-109084	CIMMYT
30	USSR-150	UAF	63	A-556	UAF	96	VL-1029	CIMMYT
31	B-34	UAF	64	A-638	UAF	97	VL-0512420	CIMMYT
32	B-34-2B	UAF	65	AES-204	UAF	98	ZL111008	CIMMYT
33	B-42	UAF	66	Antigua-1	UAF	99	VL107657	CIMMYT
						100	ZL-111040	CIMMYT

*IL, Inbred line; MRS, Maize Research Station, Faisalabad, Pakistan; MMRI, Maize and Millet Research Institute, Yousafwala, Sahiwal, Pakistan; CIMMYT, International Maize and Wheat Improvement Center, Mexico*.

## Biometrical analyses

Observations recorded on various morphological and physiological parameters were utilized in nested randomized complete block design (NRCBD) for the purpose of statistical analyses to ignore or minimize replication effects in evaluating heterogeneous segregating (BC_1_, BC_2_, and F_2_) breeding material (Snedecor and Cochran, 1989). Generation mean and variance components were analyzed in a computer program supplied by Dr. H.S. Pooni, University of Birmingham using SAS® 9.4 (SAS, 2014) Software and significance testing was done using *t*-test. The coefficients used for dissecting sum of squares (SS) of six basic generations were generated by using procedure of Little and Hills (1978).

$$\text{SS}={\left(\Sigma {\text{c}}_{\text{i}}{\text{Y}}_{\text{i}}\right)}^{2}/\text{r}\Sigma {\text{c}}_{\text{i}}{}^{2}$$

Where,

SS = Sum of squares of comparison
Σ = Summation
C_i_ = Comparison coefficients
Y_i_ = Generation totals
r = No. of replications

### Generation mean analysis (GMA)

It was performed using Mather and Jinks (1982) procedure.

$$\text{Y}=\text{m}+\alpha [\text{d}]+\beta [\text{h}]\;+\;\alpha^{2}[\text{i}]+2\alpha \beta [\text{j}]\;+\;\beta^{2}[\text{l}]$$

Where,

Y = Mean of one generation
m = Mean of all generation
d = Sum of additive effects
h = Sum of dominance effects
i = Sum of additive × additive interaction (complementary)
j = Sum of additive × dominance
1 = Sum of dominance × dominance interaction (duplicate).
α^2^, 2αβ and β^2^ are the coefficients of genetic parameters.

Means and variances of parents (P_1_, P_2_), BC_1_, BC_2_, F_1_ and F_2_ generations used in the analyses were calculated from individual plant data pooled over replications. A weighted least square analysis was also performed on generation means beginning with simplest model using parameter m only. Further models of increasing complexity (md, mdh, etc.) were fitted. The best fit model was selected when estimate of χ^2^ was non-significant with all significant parameters.

### Generation variance analysis (GVA)

Components of a genetic variance may either be additive (D), a heritable-fixable, dominance (H), a heritable-non-fixable and epistatic (E) which is non-heritable.

$$\begin{array}{l} \text{ Additive variance}(\text{D})=4\sigma^{2}{\text{F}}_{2}-2(\sigma^{2}\;{\text{BC}}_{1}+\sigma^{2}{\text{BC}}_{2})\\ \text{ Dominance variance}(\text{H})=4\sigma^{2}{\text{F}}_{2}-1/2\sigma_{\text{D}}^{2}-\sigma_{\text{E}}^{2}\\ \text{Environmental variance}(\text{E})=1/3(\sigma^{2}{\text{P}}_{1}+\sigma^{2}{\text{P}}_{2}+\sigma^{2}{\text{F}}_{1})\\ \text{ Interaction }(\text{F})=\sigma^{2}BC_{1}-\sigma^{2}{\text{BC}}_{2} \end{array}$$

Where,

F_1_ = Filial generation one
F_2_ = Filial generation two
BC_1_ = Backcross to P_1_
BC_2_ = Backcross to P_2_

A weighted least square analysis of variances was also performed (Mather and Jinks, 1982). Models incorporating E, (D and E), (D, H, and E), (D, F, and E), (D, H, F, and E) were tried. The best fit model was selected when χ^2^ was non-significant with all significant parameters.

### Narrow sense heritability (${\text{h}}_{n}^{2}$) and genetic advance (GA)

Estimates of narrow sense heritability for F_2_ generation of all traits recorded under non-stressed and heat-stressed conditions were calculated (Warner, 1952). Likewise, narrow sense heritability estimates for F_∞_ generation were also computed (Mather and Jinks, 1982).

$$\begin{array}{l} {\text{h}}_{\text{n}}^{2}{\text{F}}_{2}=0.5\text{D}/\sigma^{2}{\text{F}}_{2}\\ {\text{h}}_{\text{n}}^{2}{\text{F}}_{\infty }=\text{D}/(\text{D}+\text{E}) \end{array}$$

Where,

D = Additive variance
E = Environmental variance
σ^2^F_2_ = Phenotypic variances

Genetic advance as percent of mean (GAM%) was determined from genetic advance (GA) expressed as percentage of population mean (Allard, 1960) for all the characters under study. Expected genetic advance in next generation was calculated using the procedure of Falconer and Mackay (1996).

$$\text{GA}=\text{K}\times \sigma_{\text{p}}\times {\text{h}}_{\text{n}}^{2}$$

Where,

K = Selection differential, being 2.06 at 5% selection intensity
σ_p_ = Phenotypic standard deviation of base population
${\text{h}}_{\text{n}}^{2}$ = Narrow sense heritability of the character under selection

## Results

### Variance analyses and selection of parents

One way analysis of variance revealed statistically significant differences (*P* ≤ 0.01) among one hundred genotypes for cell membrane thermo-stability and grain yield per plant in non-stressed (NS) and heat-stressed (HS) conditions (Table 3). Likewise, two way variance analyses also suggested significant differences (*P* ≤ 0.01) among genotypes and temperature treatments under which the experiments were conducted for both cell membrane thermo-stability and grain yield per plant. Significant (*P* ≤ 0.01) interaction of genotype-temperature (G × T) suggested the existence of distinct responses among maize inbred lines for cell membrane thermo-stability and grain yield per plant under both the environments (Table 3).

**Table 3:** **Means squares of cell membrane thermo-stability and grain yield per plant under non-stressed and heat-stressed environments**.

**Parameters**		**DF**	**Cell membrane thermo-stability (%)**	**Grain yield per plant (g)**
**ONE WAY ANALYSIS OF VARIANCE**				
Genotypes	NS	99	600.68^**^	470.62^**^
	HS	99	442.50^**^	243.99^**^
Error	NS	198	1.883	35.200
	HS	198	1.702	1.322
**TWO WAY ANALYSIS OF VARIANCE**				
Genotypes (G)		99	1035.20^**^	609.30^**^
Temperature (T)		1	8233.10^**^	81739.5^**^
G × T		99	7.990^**^	105.300^**^
Error		398	1.790	20.200

*DF, Degrees of freedom; NS, Non-stressed; HS, Heat-stressed*.

** *Significant at P ≤ 0.01*.

**Figure 1:**
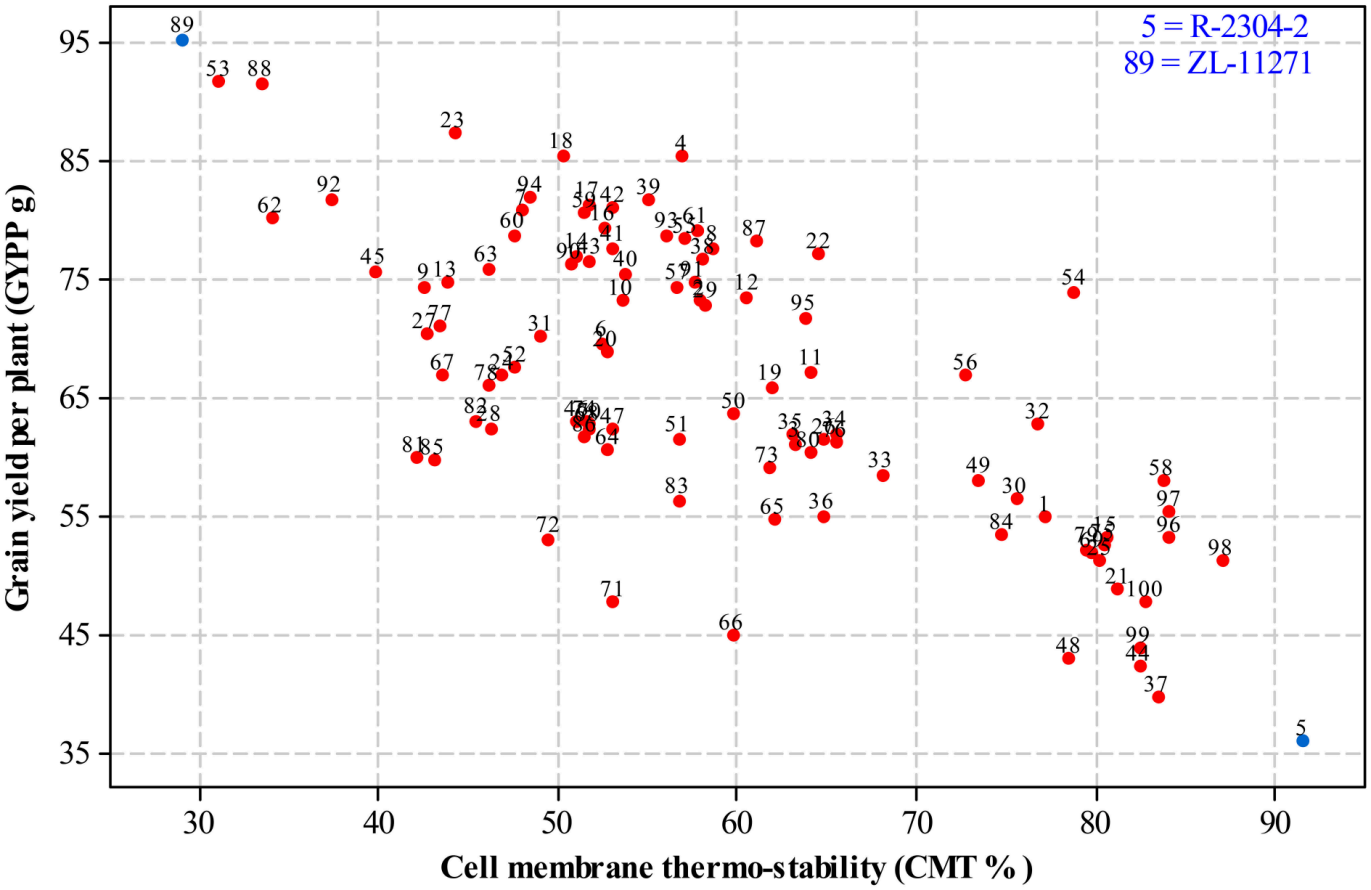
**Scatter plot of mean genotypic values for cell membrane thermo-stability (%) against grain yield per plant (g) in non-stressed conditions**.

**Figure 2:**
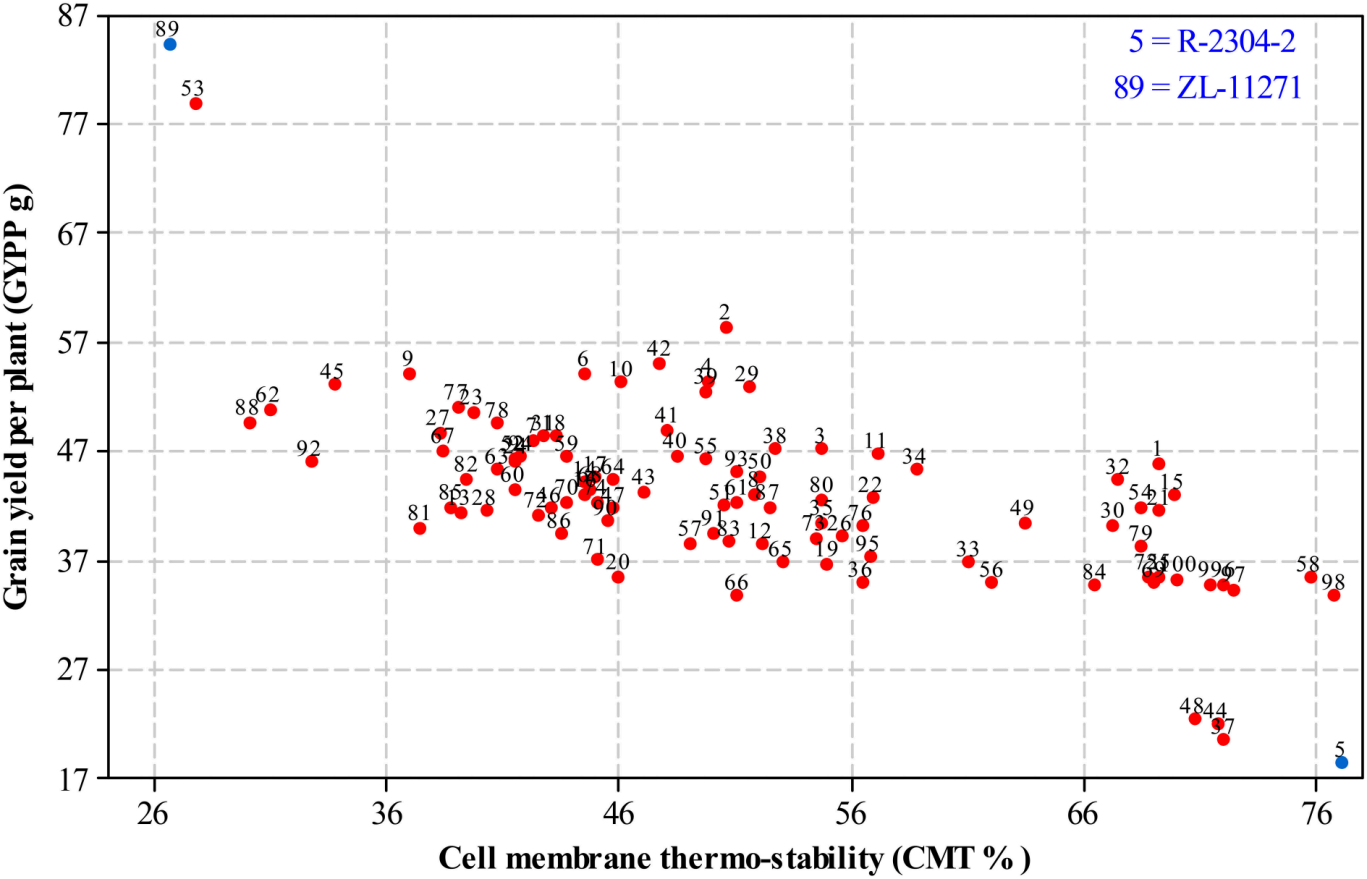
**Scatter plot of mean genotypic values for cell membrane thermo-stability (%) against grain yield per plant (g) in heat-stressed conditions**.

For selecting desired parents, scatter plots were generated by plotting mean estimates of genotypes for cell membrane thermo-stability on X-axis and grain yield per plant on Y-axis both under non-stressed and heat-stressed conditions. The graph of non-stressed regime (Figure 1) for cell membrane thermo-stability displayed only one genotype falling below 30% and one above 90% on extreme scales, however, varied numbers of genotypes were found among other scales with the highest number between scales of 50–60%. For grain yield per plant, least numbers of genotypes were recorded between extreme scales of 35 g to 45 g and 85 g to 95 g in comparison to other scales where distribution of genotypes was almost similar. Genotypes 89 and 5 were recorded at extreme scales of lowest to highest for cell membrane thermo-stability and highest to lowest for grain yield per plant, respectively in non-stressed conditions. The scatter plot exhibiting genotypic cell membrane thermo-stability in heat-stressed conditions (Figure 2) indicated that inbred lines 89 and 53 fall close to the lowest scale of 26% while inbred lines 58, 98 and 5 were found near to highest scale of 76%. Maximum genotypes fall in between 36 and 46% scale followed by 46 and 56% scale for cell membrane thermo-stability. Least numbers of genotypes were recorded for lowest scale of 17–27 g and higher scale of 77–87 g, however, more than 75 percent genotypes appeared between scales of 37–57 g regarding grain yield per plant. Similar to non-stressed conditions, genotypes 89 (ZL-11271) and 5 (R-2304-2) were recorded on extreme scales of cell membrane thermo-stability and grain yield per plant in heat-stressed conditions, therefore, selected for developing breeding material to conduct genetic studies of heat tolerance in maize.

Both these parents were used to develop six basic generations which were investigated for various traits both in non-stressed and heat-stressed environments. Significant differences (*P* ≤ 0.01) among generations, parents (P_1_ vs. P_2_) and backcrosses (BC_1_ vs. BC_2_) were observed in leaf temperature, cell membrane thermo-stability, transpiration rate, leaf firing, ear length, kernels per ear, 100-grain weight and grain yield per plant in both non-stressed and heat-stressed conditions (Table 4). Non-significant interaction of P's vs. F_1_ was observed in leaf temperature in heat-stressed and transpiration rate in non-stressed conditions. Regarding back crosses and F_2_ population interaction (BC's vs. F_2_), non-significant estimates were recorded only for leaf temperature and 100-grain weight in non-stressed and heat-stressed conditions while for transpiration rate only under non-stressed regime. Among all traits studied, the interaction of P's, F_1_ vs. BC's, F_2_ was non-significant only for transpiration rate in non-stressed and ear length in heat-stressed conditions suggesting genetic similarity in population for both these characters.

**Table 4:** **Mean squares acquired from partitioned analysis of variance of eight plant traits for six basic generations tested under contrasting environmental conditions**.

**Traits**		**Generations**	**P_1_ vs. P_2_**	**P's vs. F_1_**	**BC_1_ vs. BC_2_**	**BC's vs. F_2_**	**P's,F_1_ vs. BC's,F_2_**	**Error**
	**DF**	**5**	**1**	**1**	**1**	**1**	**1**	**10**
LT (°C)	NS	29.87^**^	129.0^**^	4.723^**^	3.103^**^	0.168^N.S.^	12.35^**^	0.136
	HS	12.10^**^	51.74^**^	0.062^N.S.^	6.232^**^	0.005^N.S.^	2.435^**^	0.044
CMT (%)	NS	1600.6^**^	5690.0^**^	973.7^**^	1062.9^**^	175.5^**^	100.6^**^	2.630
	HS	943.5^**^	3887.7^**^	624.3^**^	166.8^**^	12.19^**^	26.55^**^	0.500
TR (μ g cm^−2^ S^−1^)	NS	3.208^**^	12.96^**^	0.002^N.S.^	3.039^**^	0.008^N.S.^	0.026^N.S.^	0.009
	HS	3.930^**^	9.969^**^	4.043^**^	1.021^**^	1.292^**^	3.325^**^	0.019
LF (%)	NS	65.03^**^	182.2^**^	84.04^**^	49.27^**^	5.572^**^	4.027^**^	0.095
	HS	992.1^**^	3110.0^**^	1324.2^**^	166.9^**^	274.3^**^	85.55^**^	2.130
EL (cm)	NS	15.97^**^	10.11^**^	64.34^**^	2.400^**^	2.077^**^	0.896^**^	0.033
	HS	11.54^**^	9.077^**^	46.34^**^	1.050^**^	0.825^**^	0.397^N.S.^	0.087
KPE	NS	26784.7^**^	76297.9^**^	33385.3^**^	18967.5^**^	5090.1^**^	182.9^**^	16.50
	HS	31413.9^**^	85085.0^**^	43032.0^**^	20120.3^**^	8649.2^**^	183.0^**^	12.8
HGW (g)	NS	9.129^**^	32.34^**^	1.850^**^	4.965^**^	0.023^N.S.^	6.468^**^	0.092
	HS	11.84^**^	43.74^**^	1.534^**^	11.64^**^	0.184^N.S.^	2.084^**^	0.143
GYPP (g)	NS	2192.9^**^	5298.8^**^	4162.3^**^	723.7^**^	35.99^**^	743.7^**^	1.990
	HS	2643.5^**^	6642.9^**^	4740.1^**^	595.8^**^	59.67^**^	1179.0^**^	0.770

NS, Non-stressed; HS, Heat-stressed; DF, Degrees of freedom; NS, Non-significant; *,

** *significant at P ≤ 0.05, 0.01, respectively*.

*LT, Leaf temperature (°C); CMT, Cell membrane thermo-stability (%); TR, Transpiration rate (μg cm^−2^ S^−1^); LF, Leaf firing (%); EL, Ear length (cm); KPE, Kernels per ear; HGW, 100-grain weight (g); GYPP, Grain yield per plant (g)*.

### Genetic effects

Non-allelic epistatic digenic effects were found crucial in the inheritance of all traits under the contrasting conditions, except transpiration rate in non-stressed conditions, being under the control of additive gene action predominantly (Table 5). Both additive and dominance gene actions were reported with positive epistatic [j] and [l] interactions for leaf temperature under non-stressed while negative [j] and [l] for grain yield per plant under both non-stressed and heat-stressed conditions. Duplicate epistatic [-ve l] digenic effects were ascertained critical in the inheritance of leaf temperature under heat stress and for 100-grain weight under both the conditions. Epistatic additive [-ve d and i] and dominance [-ve h and +ve j] genetic effects were vital for cell membrane thermo-stability in distinctive temperature regimes. Complementary additive genetic effects control the inheritance pattern of transpiration rate under non-stressed conditions in comparison to heat-stressed regime where the additional dominance component was also involved. Under the contrasting temperature environments, both leaf firing and ear length were found under the influence of complementary additive with dominance genetic effects. For kernels per ear, digenic complementary gene action was recorded in non-stressed regime while both duplicate and complementary genetic effects were found imperative in heat-stressed environments.

**Table 5:** **Estimates of genetic effects and standard errors of best fitted models under contrasting conditions for various traits in a cross of *Zea mays* L**.

**Traits**		**m ± SE**	**[d] ± SE**	**[h] ± SE**	**[i] ± SE**	**[j] ± SE**	**[l] ± SE**	**χ^2^ (DF)**
LT (°C)	NS	36.4 ± 0.16^**^	−4.6 ± 0.16^**^	−7.3 ± 0.97^**^		3.1 ± 0.53^**^	5.7 ± 1.09^**^	0.55 (1)
	HS	33.0 ± 0.15^**^	−2.9 ± 0.14^**^	2.8 ± 0.98^**^			−3.0 ± 1.09^**^	2.62 (2)
CMT (%)	NS	87.7 ± 1.50^**^	−30.8 ± 0.22^**^	−49.6 ± 1.68^**^	−27.5 ± 1.53^**^	5.0 ± 1.41^**^		6.04 (1)
	HS	52.2 ± 0.32^**^	−25.5 ± 0.33^**^	−17.5 ± 0.60^**^		15.2 ± 1.53^**^		5.69 (2)
TR (μ g cm^−2^ S^−1^)	NS	1.8 ± 0.04^**^	1.5 ± 0.01^**^		0.1 ± 0.04*			1.42 (3)
	HS	3.1 ± 0.35^**^	1.2 ± 0.11^**^	4.7 ± 0.47^**^	3.3 ± 0.47^**^			2.22 (2)
LF (%)	NS	15.2 ± 0.50^**^	−5.5 ± 0.08^**^	−12.6 ± 0.54^**^	−6.1 ± 0.51^**^			0.47 (2)
	HS	37.4 ± 1.13^**^	−18.9 ± 0.38^**^	−30.8 ± 1.37^**^	−6.1 ± 1.23^**^			1.32 (2)
EL (cm)	NS	8.6 ± 0.39^**^	1.3 ± 0.08^**^	9.8 ± 0.47^**^	4.2 ± 0.40^**^			0.01 (2)
	HS	8.7 ± 0.44^**^	1.2 ± 0.10^**^	7.9 ± 0.55^**^	3.1 ± 0.46^**^			1.20 (2)
KPE	NS	104.8 ± 4.27^**^	113.0 ± 0.98^**^	239.4 ± 5.11^**^	109.0 ± 4.42^**^	9.5 ± 4.00^*^		0.21 (1)
	HS	87.9 ± 16.20^**^	118.8 ± 1.58^**^	684.2 ± 40.13^**^	261.7 ± 16.11^**^		−275.8 ± 25.10^**^	0.35 (1)
HGW (g)	NS	20.6 ± 0.13^**^	−2.3 ± 0.12^**^	5.2 ± 0.73^**^			−4.3 ± 0.79^**^	1.77 (2)
	HS	20.0 ± 0.15^**^	−2.7 ± 0.14^**^	2.9 ± 0.83^**^			−2.0 ± 0.88*	0.86 (2)
GYPP (g)	NS	65.5 ± 0.51^**^	29.7 ± 0.51^**^	68.5 ± 4.66^**^		−10.0 ± 2.71^**^	−22.9 ± 4.73^**^	3.80 (1)
	HS	51.4 ± 0.82^**^	33.3 ± 0.82^**^	79.3 ± 6.23^**^		−12.6 ± 3.74^**^	−30.6 ± 6.21^**^	3.65 (1)

*,** *Significant at P ≤ 0.05, 0.01, respectively*.

*NS, Non-stressed; HS, Heat-stressed; m, Mean; [d], Additive; [h], Dominance; [i], Additive-additive; [j], Additive-dominance; [l], Dominance-dominance; χ^2^, Chi-square; DF, Degrees of freedom; LT, Leaf temperature (°C); CMT, Cell membrane thermo-stability (%); TR, Transpiration rate (μg cm^−2^ S^−1^); LF, Leaf firing (%); EL, Ear length (cm); KPE, Kernels per ear; HGW, 100-grain weight (g); GYPP, Grain yield per plant (g)*.

### Genetic variance components

Estimates of variance components revealed greater extent and commonness of additiveness [D] in traits under study except for ear length where dominance [H] component was slightly higher under non-stressed regime (Table 6). Magnitudes of other prevailing environmental [E] and interaction [F] variances were lower than respective additive and dominance variances.

**Table 6:** **Variance components best fit models following weighted analysis, narrow sense heritability and genetic advance under contrasting conditions in a cross of *Zea mays* L**.

**Traits**		**[D] ± SE**	**[H] ± SE**	**[F] ± SE**	**[E] ± SE**	**χ ^2^ (d.f.)**	**${\text{h}}_{n}^{\text{2}}$ (%)**		**GAM%**
							***F*_2_**	***F*_∞_**	
LT (°C)	NS	21.9 ± 2.61^**^		−4.54 ± 1.87^*^	2.22 ± 0.33^**^	10.0 (3)	78.4	88.8	14.0
	HS	24.4 ± 2.75^**^			2.11 ± 0.31^**^	12.6 (4)	69.3	88.5	8.8
CMT (%)	NS	209.0 ± 18.9^**^			3.58 ± 0.53^**^	8.95 (4)	76.9	97.5	41.7
	HS	235.2 ± 22.2^**^			7.15 ± 1.06^**^	7.18 (4)	82.7	96.4	57.9
TR (μ g cm^−2^ S^−1^)	NS	0.01 ± 0.001^**^		−0.004 ± 0.001^**^	0.001 ± 0.0002^**^	11.3 (3)	92.0	95.7	110.1
	HS	6.3 ± 0.81^**^			0.83 ± 0.12^**^	0.74 (4)	68.6	86.2	53.1
LF (%)	NS	23.3 ± 2.16^**^		3.70 ± 1.53^*^	0.42 ± 0.06^**^	1.55 (3)	89.8	98.0	56.7
	HS	86.5 ± 10.1^**^			8.62 ± 1.27^**^	5.24 (4)	69.2	89.9	69.3
EL (cm)	NS	0.07 ± 0.04	0.24 ± 0.06^**^		0.02 ± 0.002^**^	8.81 (3)	88.4	95.7	48.7
	HS	13.8 ± 1.41^**^			0.76 ± 0.11^**^	4.02 (4)	73.2	92.9	34.0
KPE	NS	1468.4 ± 146.4^**^		265.2 ± 104.2^**^	62.4 ± 9.28^**^	4.28 (3)	62.0	93.0	114.8
	HS	2494.4 ± 276.4^**^			203.3 ± 30.1^**^	6.81 (4)	80.0	90.0	196.4
HGW (g)	NS	13.2 ± 1.51^**^			1.19 ± 0.18^**^	7.25 (4)	78.4	90.3	13.7
	HS	15.7 ± 1.85^**^			1.59 ± 0.24^**^	3.59 (4)	75.7	89.3	15.5
GYPP (g)	NS	822.3 ± 79.4^**^		249.9 ± 48.2^**^	17.1 ± 2.55^**^	1.87 (3)	72.2	97.0	61.4
	HS	1408.3 ± 131.4^**^			37.6 ± 5.60^**^	6.16 (4)	89.0	97.0	105.6

*,** *Significant at P = 0.05, 0.01, respectively*.

*NS, Non-stressed; HS, Heat-stressed; D, Additive; H, Dominance; F, Additive-dominance; E, Environmental. χ^2^, Chi-square; $h_{n}^{\text{2}}$, Narrow sense heritability (%); F_2_, Filial generation two; F_∞_, Filial infinity generation; GAM%, Genetic advance as percent of mean; LT, Leaf temperature (°C); CMT, Cell membrane thermo-stability (%); TR, Transpiration rate (μg cm^−2^ S^−1^); LF, Leaf firing (%); EL, Ear length (cm); KPE, Kernels per ear; HGW, 100-grain weight (g); GYPP, Grain yield per plant (g)*.

### Narrow sense heritability and genetic advance

Narrow sense heritability of F_2_ population ranged 62.0% (kernels per ear) to 92.0% (transpiration rate) in non-stressed and 68.6% (transpiration rate) to 89.0% (grain yield per plant) in heat-stressed conditions (Table 6). The magnitudes of narrow sense heritability for F_∞_ were higher than respective F_2_ population. For F_∞_ population, narrow sense heritability was low for leaf temperature (88.8%) and high for leaf firing (98.6%) in non-stressed conditions, however, in heat-stressed regime, it was the lowest for transpiration rate (86.2%) and highest for grain yield per plant (97.0%). Genetic advance as percent of mean ranged 13.7% (100-grain weight) to 114.8% (kernels per ear) and 8.8% (leaf temperature) to 196.4% (kernels per ear) in non-stressed and heat-stressed conditions, respectively. Genetic advance was low for leaf temperature in heat-stressed condition. It was moderate for 100-grain weight in both conditions while for leaf temperature in non-stressed environments. GAM% was high for cell membrane thermo-stability, transpiration rate, leaf firing, ear length, kernels per ear and grain yield per plant in both non-stressed and heat-stressed regimes.

## Discussion

Plant breeding is the art of creating new genotypes suitable for different environments. These environments may or may not be favorable for developing these varieties. Heat stress, which affects the crop production and its quality significantly, may be one of those environments. Hence, breeding for high temperature tolerance may be an effective tool for overcoming current and future environmental effects produced by global climatic changes.

In the current study, biplots for non-stressed and heat-stressed conditions suggested that ZL-11271 and R-2304-2 were the most contrasting genotypes with respect to heat tolerance among all inbred lines. These graphs also revealed a negative association between cell membrane thermo-stability and grain yield per plant in maize under both the conditions (Kaur et al., 2010; Tsimba et al., 2013). Mean estimates of cell membrane thermo-stability for all the inbred lines in heat-stressed environment were lesser in comparison to non-stressed conditions which may be due to inhibition or reduced transpiration rate under normal conditions (Akbar et al., 2009).

Generation mean and variance analyses provide information concerning genetic effects comprising additive, dominance or both additive and dominance alongwith non-allelic interactions. Such information assists in determining an appropriate breeding strategy for improving various metrical plant traits. Furthermore, this technique is also useful in measuring variance components and in assessing the nature of non-allelic interaction, which depends upon positive or negative signs of two genetic components viz., h and l. Cross combinations with either positive or negative signs on h and l components indicates presence of complementary epistasis while combinations with opposite signs on h and l components reveals duplicate epistasis (Mather and Jinks, 1982). Based on χ^2^ values, genetic models comprising two, three, four and five parameters (additive, dominance and epistatic interactions, i.e., additive-additive, additive-dominance, dominance-dominance) were found best suited to all characters under both environments. Involvement of non-allelic gene interaction in all traits corroborated the existence of potential variation for further exploitation (Iqbal et al., 2015). Both additive-dominance genetic effects were recorded for all traits under both conditions except transpiration rate under non-stressed regime which was found under the influence of only additive gene action. Negative [d] or decrease in leaf temperature, cell membrane thermo-stability, leaf firing while the increase in transpiration rate, ear length, kernels per ear and grain yield per plant in non-stressed and heat-stressed regimes may be used for developing heat resilient maize synthetics suitable for both conditions. Likewise, negative [h] or decrease in cell membrane thermo-stability and leaf firing while increase in transpiration rate, ear length, kernels per ear, 100-grain weight and grain yield per plant may be exploited for developing heat tolerant maize hybrids suitable for normal and high temperature conditions. Considering genetic and interaction effects for transpiration rate, leaf firing, ear length, kernels per ear and to some extent for cell membrane thermo-stability, simple selections or hybridization followed by either bulk or pedigree or single seed decent schemes of selection could be exercised. For leaf temperature, 100-grain weight and grain yield per plant, selections should be delayed till further appraisal in later generations to identify desirable recombinants. Both additive-dominance genetic effects had been reported in inheritance of leaf temperature (Hussain et al., 2009; Wattoo et al., 2013), cell membrane thermo-stability (Saleem et al., 2015), leaf firing (Kaur et al., 2010), ear length (Ahmed et al., 2000), kernels per ear (Muraya et al., 2006), 100-grain weight (Kumar and Gupta, 2004; Katna et al., 2005) and grain yield per plant (Aguiar et al., 2003; Muraya et al., 2006) in maize under normal and stress conditions. Non-additive genetic effects were crucial in the inheritance of transpiration rate in heat-stressed and non-stressed conditions (Akbar et al., 2009). Involvements of digenic non-allelic interactions for all traits except transpiration rate in non-stressed conditions recommended attempting multiple cross combination and establishment of large segregating plant populations for current genetic material. Further, inter-mating among desirable recombinants followed by recurrent selections would help in pooling fixable and heritable additive genes, breakage of unwanted linkages, evolution of promising transgressive segregants and exploitation of non-additive genetic effects (Singh and Pawar, 1990).

Generation variance approach had been extensively utilized by various researchers for dissecting total variability into constituent components (Azizi et al., 2006; Iqbal et al., 2015). The classification of phenotypic variance into respective genetic and environmental components is not adequate for complete knowledge of the genetic basis of any source material (Shen et al., 2011). It requires further partitioning of genetic variance into additive (D), dominance (H), environmental (E) and interaction (F) elements which is possible only through generation variance analysis. Genetic and environmental components can be estimated from experiments comprising pure genetic materials (parents, F_1_) and segregating populations (BC_1_, BC_2_, F_2_, and so on). Under heat stress conditions, only D and E variances were significant in contrast to DE, DFE and DHE components under non-stressed conditions indicating the role of environment in breeding maize for heat-stressed environments. Additive [D] variance was in greater extent than respective interaction [F] and environmental [E] components for all traits under both regimes except for ear length in non-stressed conditions where dominance [H] variance was at large. Higher magnitudes of additive variance in heat-stressed conditions pointed out its potential role in inheritance of plant traits which conferred heat tolerance in maize. Higher estimates of narrow sense heritability (F_2_ and F_∞_) and genetic advance further confirmed the role of few major genes and related genetic effects and the possibility of genetic progress of studied traits (Iqbal et al., 2015). Both heritability and genetic advance constitute an important selection criterion. Estimates of heritability and genetic advance can be classified into low (< 30%; < 10%), moderate (30–60%; 10–20%) and high (>60%; >20%), respectively (Johnson et al., 1955). Consideration of both parameters at once is more supportive in anticipating the gain under selection rather than giving due importance to anyone. A character exhibiting high heritability may not necessarily show high genetic advance and *vice versa* (Johnson et al., 1955). Estimates of narrow sense heritability for infinity generation were greater than its F_2_ generation of current genetic material which further projected its scope for genetic improvement of these characters through selections due to least genotype-environment interactions, implying that any plant breeder may perform his selections on the basis of phenotypic expression of individual plants by using simple selection procedures (Singh and Narayanan, 1993; Kant et al., 2005). Concurrent study of heritability and genetic advance (% of mean) for all traits suggested that only simple selection might be enough for further improvement of characters such as cell membrane thermo-stability, transpiration rate, leaf firing, ear length, kernels per ear and grain yield per plant in both conditions. High heritability but low to moderate genetic advance (% of mean) for leaf temperature and 100-grain weight revealed greater influence of environment in expression of these characters. Immediate selection for such traits could be misleading, therefore, required further progeny testing. Such traits can be improved by crossing potential genotypes of segregating population by means of recombinant breeding approach (Samadia, 2005). The findings and suggestions made in this manuscript belong to genetic material used herein. Further, the estimates for genetic effects, variance components and heritability could vary with methodology applied but are valid only to research materials being investigated.

## Conclusion

Results revealed that this study can be helpful to maize breeders in developing heat-resilient lines through effective selections by using reciprocal recurrent strategies for traits exhibiting both additive-dominance genetic effects. Factors like low genetic advance and polygenic nature of the trait may, however, limit realized gain from selection, therefore, required progeny testing to later generations. Testing of ZL-11271 and other potential sources in different cross combinations could help in devising broader and stronger concept of gene action controlling heat tolerance in maize.

## Author contributions

MN: Conducted the research and manuscript write-up (60%). MA: Supervised the whole process i.e., research and write-up (15%). HA: Supervised screening component (10%). MA: Facilitated in planning and conductance of research (7%). NA: Facilitated in planning and conductance of research (8%).

### Conflict of interest statement

The authors declare that the research was conducted in the absence of any commercial or financial relationships that could be construed as a potential conflict of interest.
